# The Educational Possibilities of Brieux's Drama

**Published:** 1920-02-14

**Authors:** 


### "DAMAGED GOODS" ON THE FILM.

### The Educational Possibilities of Brieux's Drama.

The importance of the moving picture both for good and for evil is one of the important factors in the social evolution of the bulk of our population. No one doubts that in this respect the film industry exercises an influence as great as that of the theatre and of literature themselves. In some ways this is unfortunate, because commercialisation is so fatally easy, so inevitable, that the maintenance of a high standard is impossible so long as the masses themselves prefer a low one; more so, unquestionably, than at the play, and very much more so than in literature. What with a voluntary censorship and with the supervision and control of the law, the film industry has definitely renounced some of the excesses which characterised it not so very long ago, but it is still too soon to rate its moral standard as equal to those of the sister arts.

### An Ethical Force.

Needless to say, Brieux's great drama, translated into English and acted here as "Damaged Goods," and now in turn made into moving pictures, is not among the undesirable class of films just alluded to. As an ethical force and a power for righteousness the play has been one of the outstanding triumphs of the theatre in this century; and the film that has been made from it is worthy of the original. To tell people that this film will do them and others good, to praise it as a moral tonic, is perhaps not the best way to induce them to visit it. Let us hasten to add that for dramatic intensity and human interest it can hold its own in any company; that it is quite sufficiently well, though not lavishly, staged and produced; and that the acting of Mr. Fisher White is superb, while the rest of the company is fully adequate.

### The Film *versus* The Stage.

The film version shows very clearly the differences between the spoken and the photographed dramatic stage; in some ways one, in some ways the other, gets the advantage. Brieux's play, depending for its propagandist effect very largely upon the enlightenment of the ignorance of its audiences, is naturally a difficult one to "film"; for the dialogue is all-important and essential. Hence, in the film version an undue proportion of explanatory labels have had to be introduced, and the "pictures" suffer accordingly. On the other hand, in the play a certain amount of the plot has to be narrated by the characters to one another; and here the film scores off the legitimate stage, for it can supplant such narrative by the representation of the actual incidents. The closing scenes of the film version are an excrescence on Brieux's plot, and independent of it; if thus artistically imperfect, they are yet very effective propaganda, and can be defended accordingly.

Mr. Fisher White's impersonation of the doctor has already been mentioned; it is easily the outstanding performance among the whole cast. His acting is not only a piece of very fine artistry; it should make any medical man proud of his profession to see its rôle thus interpreted. The "father" of the "wife", and the "mother" of the "husband" are also very well handled; the latter especially took full advantage of her numerous opportunities. Among the minor parts those of the butler and the wet-nurse seemed the most capably handled; but the company all round is a strong one, as it should be, for this is a theme not to be entrusted to bunglers.

### Some Minor Solecisms.

The management advertise the production as all-British, by which it may be supposed they mean that all the players are British. Certainly the writer of the labels is not very British; French idioms translated literally into very unidiomatic English abounded all through, to say nothing of such solecisms as Arley Street (where the "doctor" lived), which were much too frequent. Indeed, the slovenly English in which film labels are composed is one of the commonest reproaches to the picture playhouse: audiences are not very critical, presumably, so managers will not take the trouble over this ingredient of the films that they must do over the acting of the scenario, and allow the King's English to be murdered without a qualm. Two or three minor details were wrong, by the way; the "country doctor's" card (he was F.R.C.S., be it noted, and had it on his visiting-card) was such as no doctor in the Kingdom ever had printed, or ever dared to show if he did have it; and university undergraduates talking openly in broad daylight with a group of prostitutes are equally figments of the imagination as far as English universities are concerned.

Whether profit or propaganda is the main object of this film is not stated in the programme: if a guess may be hazarded from internal evidence, it is probably the former. There is, of course, nothing wrong in this; if the film cannot be made a commercial success on its intrinsic merits it will need financing somehow if it is to do any good as propaganda, so it is right and proper that it should pay its way. This it is well designed to achieve, and the more it succeeds in this sense the wider must necessarily be its educative and social influence as well. It is on show at 165 Oxford Street, W., by the W. and F. Film Service, Ltd., and will doubtless shortly be released at innumerable picture playhouses all over the Kingdom. We believe that it can do nothing but good, and hope that it will reach the hearts and minds of great numbers of spectators.

